# Anomaly Detection of Wind Turbine Driveline Based on Sequence Decomposition Interactive Network

**DOI:** 10.3390/s23218964

**Published:** 2023-11-03

**Authors:** Qiucheng Lyu, Yuwei He, Shijing Wu, Deng Li, Xiaosun Wang

**Affiliations:** 1 School of Power and Mechanical Engineering, Wuhan University, Wuhan 430072, China; 2 Hubei Key Laboratory of Waterjet Theory and New Technology, Wuhan University, Wuhan 430061, China

**Keywords:** anomaly detection, condition monitoring, interactive learning, sequence decomposition, wind turbines

## Abstract

Aimed at identifying the health state of wind turbines (WTs) accurately by using the comprehensive spatio and temporal information from the supervisory control and data acquisition (SCADA) data, a novel anomaly-detection method called decomposed sequence interactive network (DSI-Net) is proposed in this paper. Firstly, a DSI-Net model is trained using preprocessed data from a healthy state. Subsequences of trend and seasonality are obtained by DSI-Net, which can dig out underlying features both in spatio and temporal dimensions through the interactive learning process. Subsequently, the trained model processes the online data and calculates the residual between true values and predicted values. To identify anomalies of the WTs, the residual and root mean square error (RMSE) are calculated and processed by exponential weighted moving average (EWMA). The proposed method is validated to be more effective than the existing models according to the control experiments.

## 1. Introduction

The dilemma of the energy crisis and the potential of various energy sources have stimulated more advancements in renewable energy such as hydropower, solar power and wind power. Wind power, in particular, has seen a growing contribution to the global energy mix. However, due to the challenging working environment and complex conditions, WTs often incur high costs for maintenance and downtime checks [1,2,3]. Therefore, it is greatly necessary to develop an efficient and reliable method for the condition monitoring of WTs.

Anomaly detection of WTs includes the process of continuously observing if certain indicators deviate from normal behaviors [4], which can detect anomalies of WTs in the early stage [5,6]. If the indicators consistently surpass the threshold for an extended period of time, the WT will be regarded as abnormal and undergo a shutdown to check the components and resolve the malfunction.

The SCADA system captures the life-cycle information of WTs including temperature, voltage, rotation speed and vibration, as time sequences. This data is used to describe the operating condition and can be leveraged for condition monitoring by establishing a mapping between operating states and online data. Li et al., for instance, introduced a new method based on the vector autoregression (VAR) model, removing the autocorrelation so as to reduce false alarms. Additionally, a multivariate exponentially weighted moving average (MEWMA) control chart is employed to monitor the residual vector, enabling accelerated anomaly detection [7].

Deep learning methods offer the ability to optimize the parameters of the model by using back propagation algorithms, enabling robust feature extraction and nonlinear expression [8]. So, there have been quantities of research into the condition monitoring of WTs combining SCADA data with deep learning networks. Dhiman et al. formulated an anomaly-detection method for wind turbine gearboxes based on adaptive threshold and twin support vector machine (TWSVM). To minimize false alarms and missed failures, adaptive threshold-based binary classifications are proposed and proved based on measured SCADA data [9]. Addressing the issue of overfitting caused by non-interpretable model architectures, Tian et al. proposed an alternative and cost-effective methodology based on the group method of data handling (GMDH) neural network. With the automagical organization of the neural network, it averts the overfitting problems in ANN [10]. Kong et al. introduced a novel method that combines convolutional neural networks (CNN) with gated recurrent units (GRU) to enhance the accuracy of condition monitoring through spatio-temporal feature fusion [11].

As mentioned above, the anomaly detection of WTs during condition monitoring mainly depends on time series forecasting (TSF). TSF assists decision making in the foreseeing future tendency of evolution, which is generally used in scientific and engineering applications like business intelligence [12], interval forecasting [13] and price trend analysis [14]. Traditional methods like the autoregressive integrated moving average (ARIMA) model [15] and Holt–Winters seasonal method [16] pay more attention to single-variable forecasting, while lacking the applications to complex raw time series data. However, with advancements in data computing, it has been shown that new methods that address multivariate forecasting and multiple seasonalities achieve superior performance [17]. Additionally, several kinds of deep neural networks used for sequence modeling are shown to be more reliable and effective, such as temporal convolutional network (TCN) [18] and long short-term memory (LSTM) [19]. Moreover, Duan et al. proposed a graph neural network with neural Granger causality, namely CauGNN. Each variable is regarded as a graph node and each edge means the casual relationship. It is confirmed for multivariate time series forecasting in the dataset of appliances energy consumption [20].

Owing to the severe working environment of WTs, there are obvious seasonal tendencies and nonlinear characteristics in SCADA data like vibration, temperature and pressure, presenting a great challenge for prediction and anomaly detection. However, sequence decomposition makes it possible to analyze different components of the time series, including seasonality, trend and irregular signal, namely STL decomposition. By extracting the seasonal factor and mitigating the effects of seasonal fluctuations, some traditional models like RNNs are utilized to make forecasts. This approach has been successfully applied in various domains, including renewable energy generation forecasting [21], short-term load forecasting for distribution networks [22] and fault diagnosis for electro-mechanical actuators [23].

Inspired by the approaches mentioned above, we propose a novel neural network named decomposed sequence interactive network (DSI-Net) for the condition monitoring of WTs. STL is utilized to downsample the input time series to the sequences of trend and seasonality, reflecting the changing tendency and cycle features, respectively. And the interactive structure with a residual shortcutting is designed for the extraction of spatio-temporal features using CNN and GRU [24] blocks. After the extraction, we incorporate interactive learning between the two sub-sequences and the original sequence to offset the loss of information. After all the downsample-convolve-interact operations, we realign and concatenate all the low-resolution components into a new sequence representation. The main contributions are as follows:

(1) Novel models for the feature extraction with sequence decomposition: We propose a TSF framework, DSI-Net, for the condition monitoring of WTs by using the distinctive attributes of time series data from SCADA. Sequence decomposition is introduced to obtain the subsequences of trend and seasonality. By extracting and interactively exchanging spatio-temporal features, we demonstrate the effectiveness of our framework through experiments based on gearbox oil temperature (GOT) and generator bearing temperature (GBT) datasets.

(2) Interactive network structure: To compensate for the probable loss of information after downsampling, an interactive learning structure is applied in condition monitoring for the first time. The deep characteristics can be interchanged between the original sequence and subsequences, digging for the affine transformation parameters from each other.

(3) Extracting blocks for spatio-temporal features: As the extracting blocks, the architectures of CNN and GRU are introduced into an interactive learning block to establish the deep learning model for fusing spatio-temporal features in SCADA data.

The remainder of this paper is organized as follows. The theoretical track for this research is provided in Section 2. In Section 3, we expound on SCADA data and preprocessing applied in this paper. The methodologies and model structure are introduced in Section 4. The validation and results of the experiment are shown in Section 5. Ultimately, the study is concluded in Section 6.

## 2. General Flow of Condition Monitoring of Wind Turbines

Generally, the theoretical track of condition monitoring of WTs is based on TSF by extracting the sensitive monitoring indicators and the comparison with the setting alarming threshold. The indicators, such as some statistical indexes, provide insights into the operating conditions of WTs. And the alarm threshold is obtained from the historical data of WTs under different operating conditions. When the monitored indicators exceed the predefined threshold for a period, it triggers an alarm indicating an anomaly in the system.

**Figure 1:**
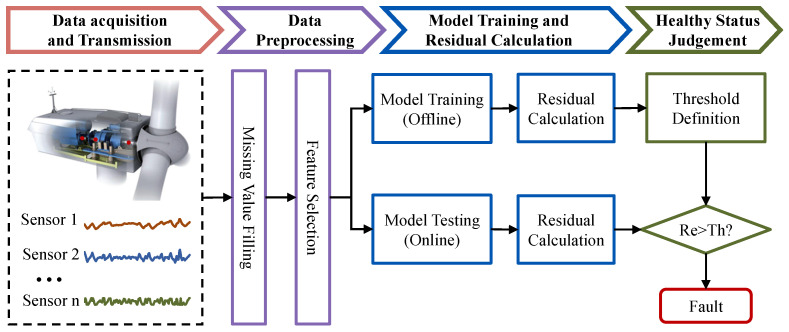
The flow of condition monitoring of wind turbines.

Based on the proposed method, the flow of condition monitoring is shown in Figure 1. In the first module, the raw data from the SCADA system are preprocessed by missing values filling, using the selection of corresponding variables to create time series datasets. The training dataset will be decomposed into subsequences according to trend and seasonality, respectively entering the model training. And then the best-trained model will be obtained. After that, the online test dataset will be fed into the model for condition monitoring. The evaluating factors and warning thresholds are calculated using EWMA to assist in discriminating abnormal conditions.

## 3. Data Preprocessing

The wind turbine gearbox used in this study has the compact mechanics of a one-stage planetary (2K-H) and two-stage parallel axis helical architecture to realize space-saving without losing the high speed-increasing ratio. There are several useless or missing data in the collected sequences because of the failure of acquisition and unscheduled downtime. So the raw data will be preprocessed first before training the model to assure accuracy.

### 3.1. Missing Value Filling

Due to the failure during the transmission and storage, some missing values and blanks break the integrality of the raw data. In order to keep the continuity in the temporal dimension of the data, it is required to fill the missing information of time sequence datasets. To retain the tendency over time, the filling values are calculated by the local mean method, shown in (1),

(1) $$x_{i}^{p}=\frac{\sum_{i=t-k-1}^{t-1}x_{i}^{p}+\sum_{i=t+1}^{t+k+1}x_{i}^{p}}{k}$$

where $x_{i}^{p}$ is the *t*-th missing value of the *p*-th time sequence variable. *k* is the length of the filling window. As for the proposed method, *k* is set to 6.

### 3.2. Selection of Corresponding Variables

The presence of redundant SCADA data can negatively impact the accuracy and efficiency of time series forecasting. To address this issue, it is important to carefully select the variables based on their relevance to the target variables. Variables that have a significant impact on the target variables should be prioritized during the model construction and training process. Statistically, the relevance of variables could be indicated by correlation analysis methods such as the Spearman correlation coefficients. It was initially proposed by Charles Spearman in 1904, and defined as a nonparametric measure of the dependence of two variables. Jan Hauke and Tomas Kossowski [25] compared the values and significance of the Pearson and Spearman correlation coefficients for the same dataset, proving the reliability of the Spearman correlation coefficients when facing the nonparametric or distribution-free problems. The Spearman correlation coefficients ρ is a nonparametric or distribution-free rank statistical metric of the strength and the direction of the random monotonic relevancy between two variables. In the gross, the Spearman correlation coefficient is simply a special modality of the Pearson coefficient, transforming the samples into ranks before calculations of correlation coefficients. But there is no need to make an assumption of linear relation and frequency profile. On the basis of the residual of two ranked variables, ρ can be expressed as [26]:

(2) $$\rho =1-\frac{6\sum d_{i}^{2}}{N(N^{2}-1)}$$

where $d_{i}=X_{i}^{\prime }-Y_{i}^{\prime }$, meaning the residual between each pair of the ranked variables. *N* is the number of samples. The positive and negative values of ρ mean the corresponding correlations. For complete monotone correlations, the absolute value is 1.

## 4. Methodology

### 4.1. Sequence Decomposition

Due to the changeable natural condition, there are plenty of instabilities in the collected SCADA data, impacting the time series forecasting severely. What is more, the general models for predicting, including RNN and Transformer, can catch the long-term dependencies of time series and extract temporal features adaptively. However, when facing the lack of abundant training data or lopsidedness, the deep learning network may be overfitting with a poor generalization performance. Therefore, to tackle this dilemma, this paper utilizes a sequence decomposing block as an inner operation, enabling the extraction of the long-term stationary features from predicted intermediate hidden variables stage by stage. Virtually, as a classic method in signal processing [27], the analysis of multi-resolution has been used in time series processing like classification [28] tasks and so on. In this paper, specifically, we adapt the moving average to smoothen periodic fluctuations and emphasize the long-term trends. For input sequence $X\in R^{L\times d}$, the calculations [29] are shown as below.

(3) $$\begin{array}{cc} X_{padding}= & Padding(X) \end{array}$$

(4) $$\begin{array}{cc} \;\;\;\;X_{t}= & AvgPool(X_{padding}) \end{array}$$

(5) $$\begin{array}{cc} X_{s}= & X-X_{t} \end{array}$$

where $X_{padding},X_{s},X_{t}\in R^{L\times d}$ denote the sequences after padding, seasonality subsequences, and trend subsequences, respectively. *L* and *d* are set to 32 and 12 in this paper. The AvgPool(∘) is utilized to extract trend information in the sequences. In conclusion, the above calculations are summarized as $X_{s},X_{t}=SeriesDecomp\;\left(X\right)$ in the block of sequence decomposition.

### 4.2. Interactive Learning

**Figure 2:**
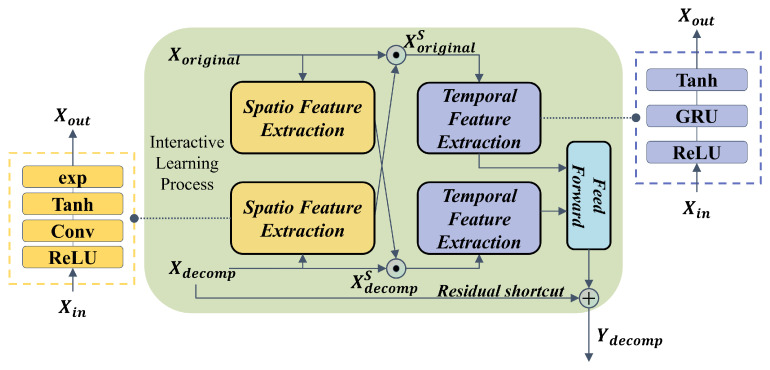
The structure of the interactive learning block.

After dividing the raw data into sequences of trend and seasonality, the component with high predictability can be insulated from the periodic effect and fluctuant noise. But some drawbacks also cannot be neglected because the decomposed subsequences usually have a similar tendency on both sides of a time point. So, we introduce an interactive learning structure combined with sequence decomposition to dig the characteristics thoroughly. Liu et al. [30] proposed sample convolution and interaction network (SCINet) for the time series forecasting task, which uses an interactive learning method to achieve high-precision forecasts on various time series datasets. Inspired by their work, this paper raised a novel interactive learning structure combined with spatio and temporal feature extraction methods, which is shown in Figure 2.

The decomposed sequence and the original sequence are input to the spatio feature extraction module simultaneously, enabling the model to capture the underlying features through the interactive learning process between two sequences. After being pre-activated by the ReLU function, various spatio features are extracted with assigned weights in the one-dimensional convolutional layer. And the hyperbolic tangent (Tanh) is deployed to make a nonlinear mapping as an activating function. As mentioned above, the spatio feature extraction can be formulated as:

(6) ς(x)=Tanh(Conv(ReLU(x)))

Moreover, interactive dot multiplication is introduced as a step of interactive learning. After the operation of an exponential (exp) function, the output sequences of spatio feature extraction are guaranteed to be positive vectors, which are then entered into the interactive dot multiplication. The decomposed sequences $X_{decomp}$ are multiplied with the result of the original sequence $X_{original}$ from CNN and obtain the feature vector with an unaltered length. Correspondingly, the original sequence $X_{original}$ also undergoes the same interactive operation to learn the extracted feature mutually so as to mine for the hidden information. The process is shown as:

(7) $$\begin{array}{c} X_{original}^{s}=X_{original}•\exp\left(\varsigma \left(X_{decomp}\right)\right) \end{array}$$

(8) $$\begin{array}{c} X_{decomp}^{s}=X_{decomp}•\exp\left(\varsigma \left(X_{original}\right)\right) \end{array}$$

where • means the dot product operation. After that, the results of the interactive process will enter the GRU module for temporal feature extraction. As one of the variants of LSTM, gated recurrent unit (GRU) makes a simplification of gates, enabling the enhancement of efficiency while maintaining excellent temporal feature capture. And the feed-forward layer is set with two fully connected layers, making the high–low dimension transformation, where two results of temporal feature extraction modules are multiplied. In particular, the shortcut connection introduces $X_{decomp}$ to the adding operation instead of dot multiplication, where the residual architecture lowers the risk of overfitting. The formulas are as follows:

(9) $$\begin{array}{cc} \psi (x)= & \mathrm{Tanh}(\mathrm{GRU}(\mathrm{ReLU}(x))) \end{array}$$

(10) $$\begin{array}{cc} X_{decomp}^{f}= & \psi \left(X_{original}^{S}\right)•\psi \left(X_{decomp}^{S}\right) \end{array}$$

(11) $$\begin{array}{cc} Y_{decomp}= & X_{decomp}^{f}+X_{decomp} \end{array}$$

where ψ expresses the temporal feature extraction process. $X_{decomp}^{f}$ is the output of the temporal feature extraction module. $Y_{decomp}$ is the final output of the interactive learning block. In summary, the above equations are summarized as $Y_{decomp}=InterLearning\left(X_{decomp}\right)$ as the interactive learning process.

### 4.3. DSI-Net

**Figure 3:**
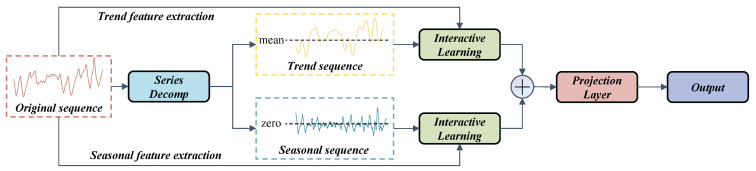
The structure of DSI-Net.

Based on the mentioned sequence decomposition and interactive learning, the novel DSI-Net is established as shown in Figure 3.

There are three main blocks in the network, namely SeriesDecomp, InteractiveLearning and ProjectionOutput. The original sequence is decomposed in the first block, with the subsequences entering feature extractions of trend and seasonality. The extractions after division contribute to the interpretability and high precision of the predicting model. To improve the robustness of the network, the first and last points of the time series are duplicated. This duplication ensures that the sequences have the same dimensions and helps maintain consistency throughout the analysis.

Two subsequences $X_{original}$ and $X_{decomp}$, can interact with the original time series in the block of interactive learning respectively so as to acquire the spatio-temporal features hidden in the deep-seated scale. Two interactive sequences engage mutually between CNN module and GRU module, which are used for spatio and temporal feature extractions correspondingly. After the output of the feed-forward layer, a skipping connection, namely residual connection [31], is added to prevent overfitting.

After interactive learning, the output $Y_{decomp}$ will be added together and fed into the projection layer, where the eigenvector can be mapped from the primordial dimension to the required dimension adapting to the condition monitoring task through the convolutional layer with a kernel size of 1. The process of the projection layer is represented below.

(12) $$\begin{array}{c} Y_{t}=Y_{seasonality}+Y_{trend} \end{array}$$

(13) $$\begin{array}{c} Y=Projection(Y_{t}) \end{array}$$

where $Y_{seasonality}$ and $Y_{trend}$ are the output of the seasonal and trend feature extraction modules, respectively. $Y_{t}$ represents the sum of them, and $Y\in R^{L_{out}\times d}$ denotes the output of the DSI-Net, in which $L_{out}$ and *d* are both set to 12 in this paper.

### 4.4. General Procedures of the DSI-Net

**Figure 4:**
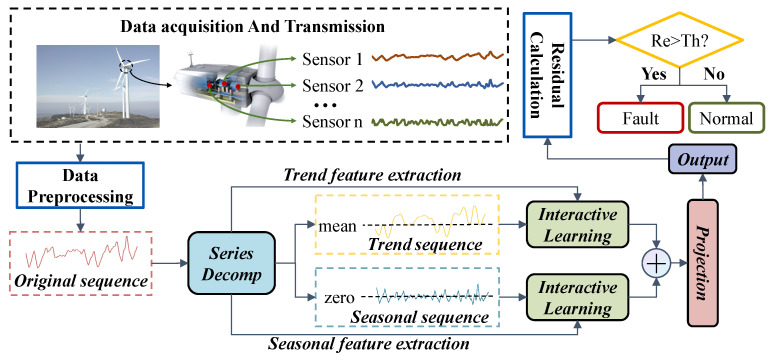
General procedures of the DSI-Net.

The DSI-Net is developed for the condition monitoring of WTs. As shown in Figure 4, the general procedures of the DSI-Net are shown below.

Step 1: Collect data from the SCADA system.

Step 2: Preprocess the data and divide them into training, validation and test datasets.

Step 3: Decompose the sequences into trend sequences and seasonality sequences by SeriesDecomp block.

Step 4: Extract underlying spatio and temporal features through the InteractiveLearning block.

Step 5: Sum the outputs of the trend and seasonal feature extraction modules and project the vector to the required dimension.

Step 6: Calculate the residual and judge the working condition of WTs.

## 5. Validation

### 5.1. Dataset Description

**Figure 5:**
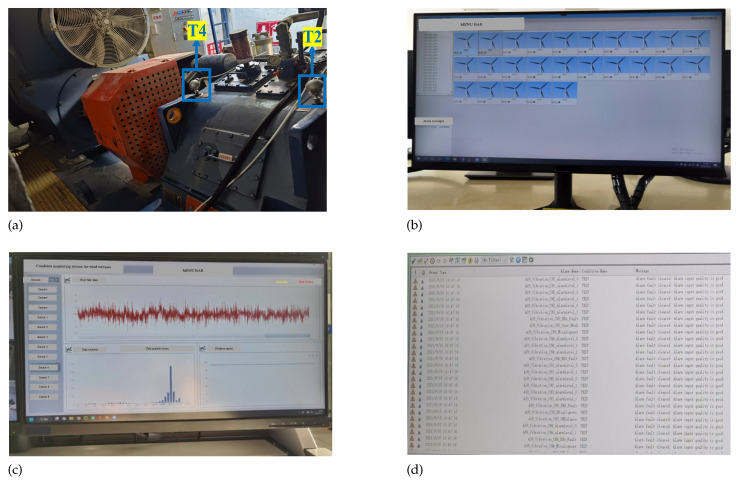
Pictures of the monitoring system and sensors in the field: (**a**) the installation position of certain sensors, (**b**) home page of the system, (**c**) data visualization and processing page, (**d**) fault pre-warning and record page.

The SCADA data used in this paper were collected from one wind farm located in Hubei, China, with records of the operation state of the No. 2, No. 19 and No. 22 WTs from January 2021 to November 2021. The dataset contains temperature data from 14 measure points, as shown in Table 1. The sample interval is set as 10 min. The variable names and their corresponding abbreviations are shown in Table 1. And the on-site pictures of the condition monitoring system for WTs are shown in Figure 5, including the picture of the installation position of certain sensors (Figure 5a), home page of the system (Figure 5b), data visualization and processing page (Figure 5c) and fault pre-warning and record page (Figure 5d).

### 5.2. Data Preprocessing

**Figure 6:**
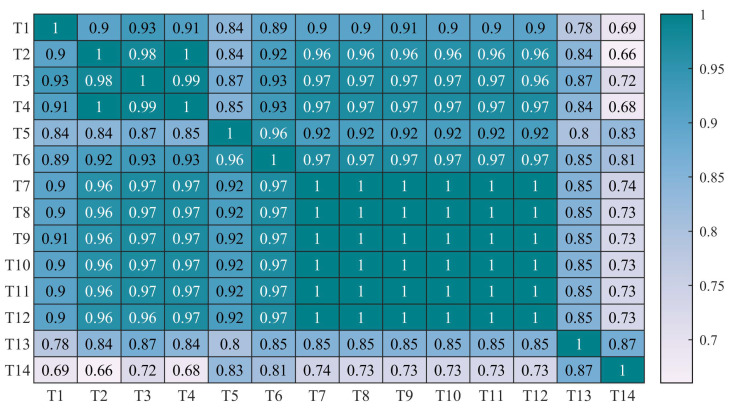
The heatmap of the Spearman correlation coefficients.

To advance the convergence rate and accuracy of the deep learning model, data preprocessing methods including missing values filling and selection of corresponding variables are necessary and vital. As mentioned in Section 3, the method of the Spearman correlation coefficients is utilized in this paper to select the features that are of great influence on the status of WTs, and the heatmap of the coefficients is shown in Figure 6. The observations indicate that T13 and T14 have few correlations with the working condition of WTs. Therefore, the input eigenvector can be selected and expressed by X=[T1,T2,T3,T4,T5,T6,T7,T8,T9,T10,T11,T12].

### 5.3. Experiments Based on the DSI-Net

In this research, contrast experiments based on different WTs are formulated to verify the capability of prediction both in accuracy and in generalization. According to the running log, there was no over-temperature record of the No. 2 WT. The GOT of the No. 19 WT alarmed on 13 May, and the GBT of the No. 22 WT alarmed on 14 June. Therefore, the data of the No. 2 WT from January to August are chosen as the training dataset (34,992 pieces of data). The validation is based on the datasets of the No. 2 WT in Sep (4320 pieces of data). The data of the No. 2 and No. 19 WTs in October serve as the test datasets (8928 pieces of data).

#### 5.3.1. Validation of the Data Preprocessing Method

**Figure 7:**
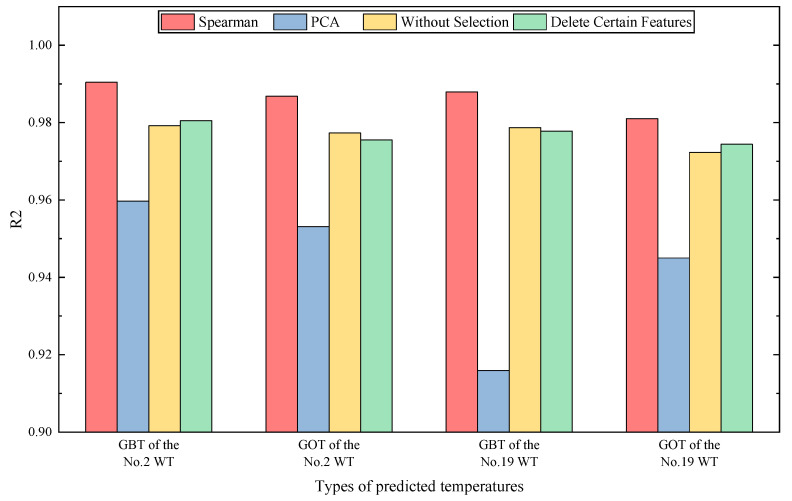
Comparison of the data preprocessing methods for DSI-Net: (red) the Spearman method, (blue) the PCA method, (yellow) without feature selection, (green) delete the features T8–T12.

Data preprocessing methods have a significant impact on the convergence of the deep learning models: some control experiments based on DSI-Net are utilized to verify the Spearman feature selection method. Principal Component Analysis (PCA) [32] is a feature dimension reduction method that is widely used for data preprocessing, but mainly for tasks with plenty of features. It can be observed in Figure 6 that the Spearman coefficient between some features is 1, indicating a high correlation. While enhancing the diversified expression of health information, these features also pose a risk of feature redundancy. So experiments that delete the features T8–T12 are conducted to validate its effectiveness. The experiment without feature selection or feature dimension reduction is also carried out, and the results are presented in Figure 7. It can be analyzed that the Spearman feature selection method achieves the highest prediction accuracy compared to other experimental groups, which validates the effectiveness of the feature selection method. The R2 of the experimental group using the PCA method is significantly less than other groups. The limitations of the PCA method include its ability to only handle linear relationships between features and the potential for information loss during the reduction process. Given the complexity of the relationships between SCADA data and the limited features available, PCA may not be the optimal method.

#### 5.3.2. Validation of Effectiveness of Blocks in the DSI-Net

As mentioned above, there are two feature extraction modules based on CNN and GRU in interactive learning: digging out the hidden features both in spatio and temporal dimensions, respectively. The inner operation of the sequence decomposing block assists in learning the complex temporal patterns in long-term forecasting context in the interactive learning block. The contrast experiments based on healthy data are utilized to further verify the effectiveness of the interactive learning and the sequence decomposition blocks in the DSI-Net.

**Figure 8:**
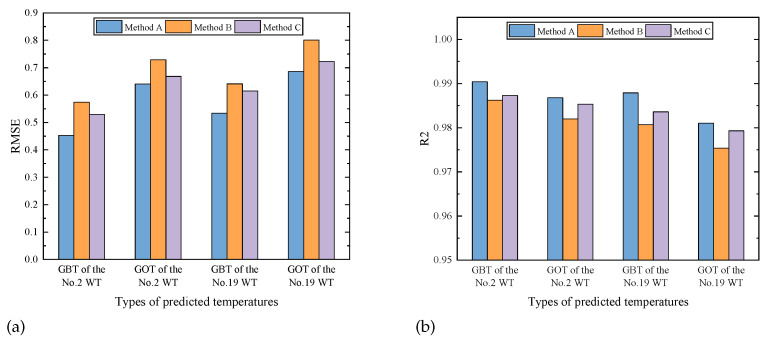
Comparison of prediction accuracy of different feature extraction methods in the DSI-Net: (**a**) the RMSE value, (**b**) the R2 value.

For the GOT and GBT datasets from No. 2 and No. 19 WT, a comparison of three different feature extraction methods in the DSI-Net for spatio–temporal features, spatio features and temporal features, namely Method A, B and C, respectively, is shown in Figure 8. Obviously, due to the fusion of the spatio and temporal information through the interactive learning process, the proposed method obtains a better performance than others.

**Figure 9:**
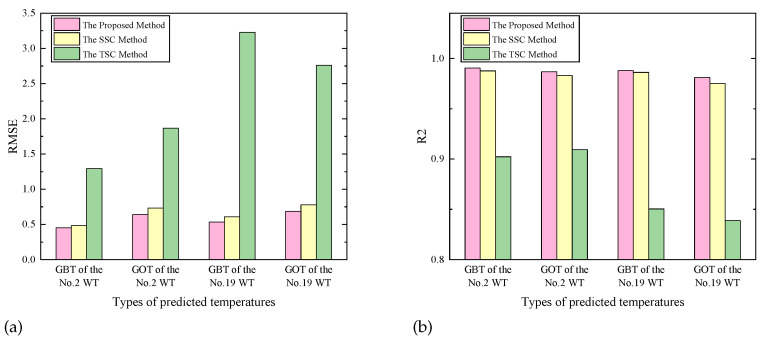
Comparison of prediction accuracy of different decomposition methods in the DSI-Net: (**a**) the RMSE value, (**b**) the R2 value.

The effectiveness of the trend-seasonal decomposition can be verified, as shown in Figure 9. It turned out to be valid that the proposed method has a more outstanding accuracy with a lower RMSE value and a higher R2 value compared with the methods making seasonal subsequences constant (the SSC method) or making trend subsequences constant (the TSC method). It is worth noting that the accuracy of the DSI-Net only slightly decreases when forbidding the decomposed seasonal sequence, rather than a dramatic decline occurring when forbidding the decomposed trend sequence. This can be explained by the fact that the trend information of a sequence plays a more important role in the prediction process, predicting the overall trend of the eigenvalues. Based on it, the seasonal information further enhances the accuracy of the prediction. The DSI-Net effectively combines these two pieces of information, resulting in improved performance.

#### 5.3.3. Validation of Healthy Data

**Figure 10:**
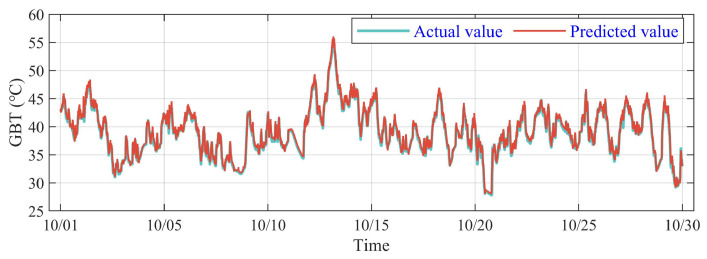
Comparison of predicting and actual value for GBT on the No. 2 WT.

The validation is based on the test dataset from the No. 2 WT, and the actual and predicted value curves of GBT are shown in Figure 10. It is illustrated that two curves coincide approximately, demonstrating the outstanding performance of the DSI-Net on the predicting task. It is worth noting that the temperature fluctuates speedily on 13 October, 22 October and 29 October, which means instability for the prediction model. However, the trained DSI-Net fits the sharp change that appeared in the actual curve primely, verifying the advantageous extraction of the spatio and temporal features using DSI-Net.

**Figure 11:**
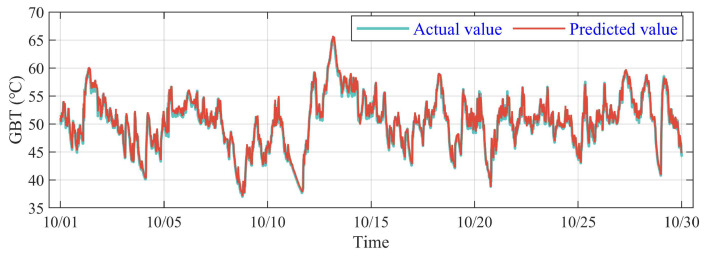
Comparison of predicting and actual value for GOT on the No. 2 WT.

Analogously, DSI-Net has an excellent performance on the predicting task of GOT and fits the actual curve well, which is shown in Figure 11. In general, DSI-Net has an outstanding predicted ability on different temperatures of WTs.

#### 5.3.4. Validation of Anomaly Detection

To further verify the robustness of the proposed method, the abnormal data are input to the trained DSI-Net, including the GBT with anomaly of the No. 22 WT from 12 June to 15 June and the GOT with an anomaly of the No. 19 WT from 12 May to 15 May. The results of the prediction cannot reflect the condition visually. So we introduce residuals between the true values and the predicted values to represent the healthy status of WTs. In this paper, the root mean squared error (RMSE) is selected to process the residual for a more stable value, which can be expressed as [33].

(14) $$RMSE_{t}=\sqrt{\frac{1}{k}\sum_{i=t-k}^{t}{\left({\hat{y}}_{i}-y_{i}\right)}^{2}}$$

where *k* is the length of RMSE window, which is set as 6. And $y_{i}$, ${\hat{y}}_{i}$ denote the true and predicted values respectively at the point *i*.

Meanwhile, the exponentially weighted moving averages (EWMA) method [34] is applied to smoothen the curve while keeping a great sensitivity to extremely small changes, which can be calculated by

(15) $$RES_{t}=\lambda RMSE_{t}+\left(1-\lambda \right)RES_{t-1}$$

where *t* is the time step and $RMSE_{t}$ is the RMSE value at the time *t*. λ is a constant from 0 to 1, reflecting the effect from the prior data. $RES_{0}$ is initially the mean of the output residual and the $RES_{t}$ is the result of EWMA. Then, the EWMA control chart is utilized to determine the upper control limit (UCL) for condition monitoring. And the warning threshold is calculated as follows [11]:

(16) $$UCL\left(t\right)=\mu_{e}+K\sigma_{e}\sqrt{\frac{\lambda }{2-\lambda }\left[1-{\left(1-\lambda \right)}^{2t}\right]}$$

where $\mu_{e}$ and $\sigma_{e}$ are the mean and standard deviation of RES of the training data. *K* and λ are parameters that control the range of the threshold.

**Figure 12:**
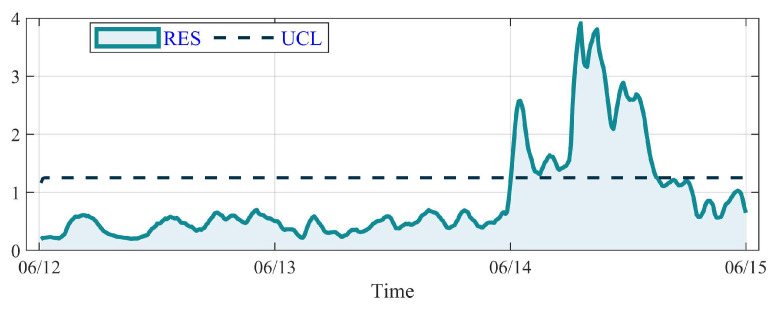
The RES curve (after EWMA) of GBT on the No. 22 WT.

The result of GBT with a RES curve and the UCL is shown in Figure 12. The average value of RES for GBT in the healthy period is 0.67 and the UCL is 1.25. It is illustrated that the RES value fluctuates slightly under the UCL on 12 June and 13 June. But an abrupt peak appears sharply at the end of 13 June and exceeds the UCL at 0:00, 14 June. Then it fluctuates above the UCL for a while and falls down to the UCL at 14:50, 14 June, regarded as abnormal. According to the running log of the No. 22 WT, the anomaly of GBT first appeared at 7:10, and the overtemperature alarm is removed at 11:10. It is worth noting that the DSI-Net detects the GBT anomaly and sets off an alarm 7.2 h in advance with great reliability.

**Figure 13:**
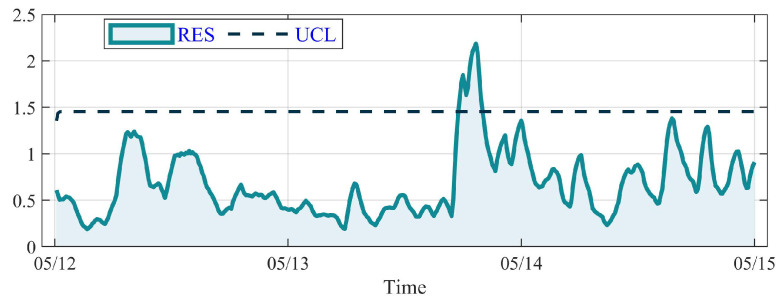
The RES curve (after EWMA) of GOT on the No. 19 WT.

The GOT monitoring result with a RES curve and the UCL are shown in Figure 13. The average value of RES for GOT in the healthy period is 0.71 and the UCL is 1.45. From 12 May to 14 May, it can be observed that the abnormal change comes into being when the RES value goes beyond the UCL at 13:30, 13 May and declines at 16:00, 13 May. According to the operating log of the No. 19 WT, it can be indicated that the anomaly of GOT occurred between 14:10 and 15:40. The result of experiments shows that the proposed method monitors the anomaly of GOT effectively and alerts in advance.

As mentioned above, with the assistance of sequence decomposition, the proposed method has a supreme ability in component extraction focused on more predictable ingredients. Additionally, the structure of interactive learning enables the identification and extraction of hidden features by leveraging similar trends from both sides of a single point. The great sensitivity to characteristic changes enhances the accuracy of anomaly detection with the implementation of DSI-Net.

### 5.4. Comparative Study of Models

The proposed model obtains further validation based on comparisons between the DSI-Net and a variety of typical models such as GRU [35], LSTM [36], CNN-LSTM [37], and SCI-Net [30].

#### 5.4.1. Validation of Healthy Data

Quantitatively speaking, the mean absolute error (MAE), mean square error (MSE), root mean square error (RMSE) and determination coefficient ($R^{2}$) are applied as the evaluating index to assess the ability of the models for condition monitoring. The dataset of verification is from SCADA of the No. 2 and No. 19 WTs in September. And the parameter configuration and operating environment of all comparative experiments are consistent.

The results based on healthy GBT and GOT are shown in Table 2 and Table 3, respectively. The DSI-Net has demonstrated excellent performance in forecasting tasks for both the No. 2 and No. 19 WTs. It outperforms other models with higher $R^{2}$ and lower MAE, MSE, and RMSE. When validating the data from the No. 19 WT by using the trained model based on the No. 2 WT, the evaluating indicators experience a little degradation due to variations in the installation sites of sensors, location of WTs, working condition of WTs, etc. The residual connection added in the interactive learning block enables avoiding overfitting, which boosts the accuracy compared with other existing models.

#### 5.4.2. Validation of Anomaly Detection

**Figure 14:**
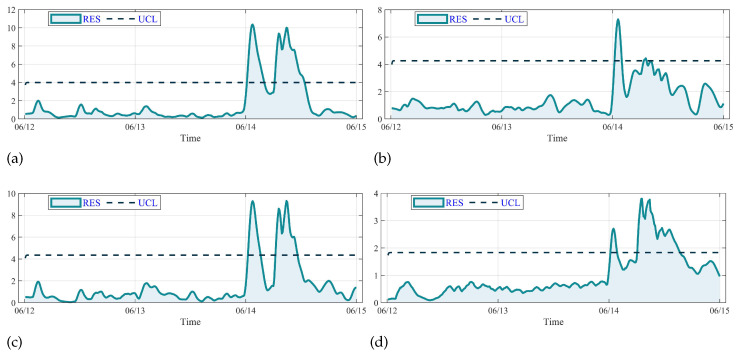
Comparison of the GBT monitoring results: (**a**) the result of GRU, (**b**) the result of LSTM, (**c**) the result of CNN-LSTM, (**d**) the result of SCI-Net.

The GBT monitoring results of control models are shown in Figure 14. As shown in Figure 14a,c,d, it can be seen that curves of GRU, CNN-LSTM and SCI-Net are similar, in which the RES value exceeds the UCL at around 0:00, 14 June and falls below the UCL at around 3:00, 14 June. After an undulation under the UCL, the RES value goes over the line again at about 7:50, 14 June. But according to the operating log, the GBT anomaly occurred between 7:10 and 11:10. The GBT monitoring results of GRU and CNN-LSTM can be regarded as false alarms. The GBT monitoring results of LSTM shown in Figure 14b indicates that it alarms between 0:10 and 1:50. The RES value fluctuates in a small range above the UCL at 6:40 and falls below it rapidly, indicating there is no anomaly on WTs. The monitoring result of LSTM is not consistent with the operating status of the No. 22 WT, which shows poor dependability.

**Figure 15:**
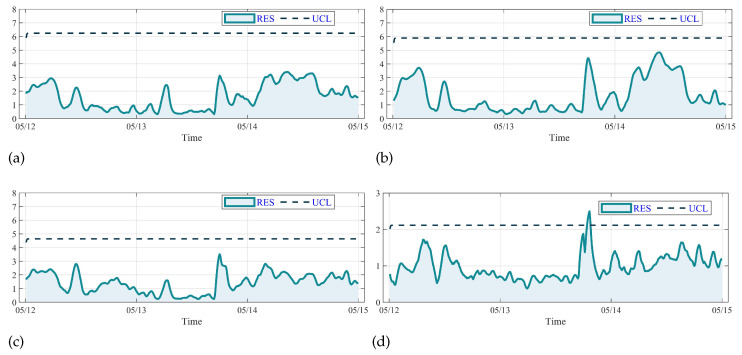
Comparison of the GOT monitoring results: (**a**) the result of GRU, (**b**) the result of LSTM, (**c**) the result of CNN-LSTM, (**d**) the result of SCI-Net.

The GOT monitoring results of GRU, LSTM and CNN-LSTM shown in Figure 15a–c indicate that the RES value is under the UCL from 12 May to 14 May without fault alarm. However, according to the working log, the GOT anomaly from 14:10 to 15:40 is not detected. The result of SCI-Net in Figure 15d shows that the detection of anomaly has a great delay of several hours. Moreover, owing to the lack of predicting accuracy, the threshold value of GRU, LSTM and CNNLSTM is larger than that of SCI-Net and DSI-Net. This instability in condition monitoring of wind turbines is attributed to the shallow structures of these models, which fail to effectively extract underlying features from the SCADA data, resulting in poor prediction accuracy. On the other hand, the SCI-Net has an unsatisfactory performance on the GBT monitoring task. Due to the stacked interactive learning process of two split sequences, the SCI-Net achieves a higher predicting accuracy and lower threshold of RES compared with other mentioned models. But it only pays attention to digging out the temporal information hidden in the sequence, which results in inadequate usage of the spatio and temporal features and the abnormal fluctuation of RES value.

Differing from the above-mentioned, the proposed DSI-Net shows a splendid performance on the condition monitoring of WTs, especially on anomaly detection. The decomposition of trend and seasonality helps to focus on the spatio-temporal features. Meanwhile, the original and decomposed sequences, which are similar in terms of the time step, make interactive learning take full advantage of the extracted features. The results confirm its effectiveness.

## 6. Conclusions

In this paper, a DSI-Net model is proposed and applied for the condition monitoring of WTs by predicting the tendency of GOT and GBT. Compared with the existing models, the proposed model enhances the accuracy and capability of anomaly detection. The conclusions are as follows:

Firstly, a TSF framework, DSI-Net, is proposed for the condition monitoring of WTs based on SCADA data. Especially, we take sequence decomposition as one of the preprocessing methods to create the subsequences of trend and seasonality, which can dig out the hidden features from spatio and temporal dimensions. After that, to compensate for the possible loss after down sampling of data, an interactive learning structure is utilized for feature extraction. A continuous interchange of features between the original sequence and subsequences enables the exploration of the affine transformation parameters.

For the inner structure of the interactive learning block, we choose CNN and GRU as the extracting units to establish the deep learning model to fuse spatio-temporal features in SCADA data. Additionally, to enhance the generation ability of the model and avoid overfitting during the training, a residual connection is also introduced in the final step of the interactive learning block. What is more, based on the raw data from the No. 2 and No. 19 WTs of a wind farm in Hubei, China, the experiments focus on TSF tasks of gearbox oil temperature and generator bearing temperature datasets. The proposed model is verified with higher accuracy and a superior generalization compared with other existing models. Ultimately, based on datasets including anomalies, contrast experiments between DSI-Net and other existing models are listed. With the advancement in sequence decomposition and interactive learning module, the DSI-Net model presents excellent feasibility on the condition monitoring of WTs.

This paper focuses on the condition monitoring of WTs. The SCADA system and monitoring system mentioned above are actually insulated from the Internet. However, the monitoring results will be severely influenced if the data are disturbed by a malicious attack from the Internet, so the network safety of monitoring systems will be a potential research direction. Additionally, following the existing research, fault classification and grading will also be the focus of future research, which is of great significance to the smart operation of WTs. 

## Figures and Tables

**Table 1:** Sample data of the SCADA system.

No.	Variable Name	No.	Variable Name
T1	Gearbox inlet oil temperature	T8	Generator winding U2 temperature
T2	Gearbox input shaft temperature	T9	Generator winding V1 temperature
T3	Gear oil temperature	T10	Generator winding V2 temperature
T4	Gearbox shaft temperature	T11	Generator winding W1 temperature
T5	Generator bearing A temperature	T12	Generator winding W2 temperature
T6	Generator bearing B temperature	T13	Main bearing gearbox side temperature
T7	Generator winding U1 temperature	T14	Main bearing rotor side temperature

**Table 2:** The results of the comparative experiments based on healthy GBT data.

Model	Evaluation Indicators							
	MAE		MSE		RMSE		$R^{2}$	
	No. 2	No. 19	No. 2	No. 19	No. 2	No. 19	No. 2	No. 19
GRU	0.7395	1.4893	1.2894	2.9295	1.1355	1.7115	0.9688	0.9018
LSTM	0.9484	1.6118	1.6777	3.4010	1.2952	1.8441	0.9594	0.8860
CNN-LSTM	1.0116	1.0546	1.8194	1.8230	1.3488	1.3502	0.9560	0.9389
SCI-Net	0.5731	0.7697	0.4706	0.7943	0.6860	0.8912	0.9725	0.9733
DSI-Net	0.3728	0.4442	0.2053	0.2852	0.4531	0.5340	0.9904	0.9879

**Table 3:** The results of the comparative experiments based on healthy GOT data.

Model	Evaluation Indicators							
	MAE		MSE		RMSE		$R^{2}$	
	No. 2	No. 19	No. 2	No. 19	No. 2	No. 19	No. 2	No. 19
GRU	1.9581	1.3458	5.2674	3.0874	2.295	1.7571	0.9211	0.9135
LSTM	1.5600	1.4737	3.8110	4.6292	1.9526	2.1515	0.943	0.8704
CNN-LSTM	1.4312	1.3580	2.8642	2.8949	1.6924	1.7014	0.9571	0.9189
SCI-Net	0.7892	0.8255	0.8725	1.0011	0.9341	1.0005	0.9596	0.9719
DSI-Net	0.5180	0.5642	0.4099	0.4710	0.6402	0.6863	0.9868	0.9810

## Data Availability

Not applicable.
